# Photonic bandgap microcombs at 1064 nm

**DOI:** 10.1063/5.0191602

**Published:** 2024

**Authors:** Grisha Spektor, Jizhao Zang, Atasi Dan, Travis C. Briles, Grant M. Brodnik, Haixin Liu, Jennifer A. Black, David R. Carlson, Scott B. Papp

**Affiliations:** 1 Time and Frequency Division, National Institute of Standards and Technology, Boulder, Colorado 80305, USA; 2 Department of Physics, University of Colorado, Boulder, Colorado 80309, USA; 3 Octave Photonics, Louisville, Colorado 80027, USA

## Abstract

Microresonator frequency combs and their design versatility have revolutionized research areas from data communication to exoplanet searches. While microcombs in the 1550 nm band are well documented, there is interest in using microcombs in other bands. Here, we demonstrate the formation and spectral control of normal-dispersion dark soliton microcombs at 1064 nm. We generate 200 GHz repetition rate microcombs by inducing a photonic bandgap of the microresonator mode for the pump laser with a photonic crystal. We perform the experiments with normal-dispersion microresonators made from Ta_2_O_5_ and explore unique soliton pulse shapes and operating behaviors. By adjusting the resonator dispersion through its nanostructured geometry, we demonstrate control over the spectral bandwidth of these combs, and we employ numerical modeling to understand their existence range. Our results highlight how photonic design enables microcomb spectra tailoring across wide wavelength ranges, offering potential in bioimaging, spectroscopy, and photonic-atomic quantum technologies.

Microresonator frequency combs have emerged as a transformative technology,^1,2^ enabling many applications^3^ due to their unique properties. These properties encompass a wide repetition frequency range from gigahertz to terahertz, octave-spanning bandwidth,^4^ and the potential for seamless integration and large-scale fabrication.^5^ Notably, microcombs can exhibit high conversion efficiency^6,7^ and even achieve nearly lossless efficient operation^8^ when paired with photonic-crystal gratings.^9–11^ Their high spectral coherence properties^12^ further enhance their applicability in various domains, ranging from data communication^13–17^ through spectroscopy^18–20^ to frequency synthesis.^21^

One of the pivotal aspects of microresonator frequency combs is the ability to design and manipulate factors influencing nonlinear propagation like group-velocity dispersion (GVD, hereafter dispersion). Introducing non-perturbative couplings that favor the emergence of novel nonlinear eigenstates can further refine and tailor microcombs for specialized applications.^10,22^ The intrinsic wavelength independence of the Kerr effect, which facilitates nonlinear wavelength conversion in microcombs, provides an edge in frequency-comb generation. While soliton microcombs at 1550 nm have been extensively studied, leveraging the mature properties of that band, there is a growing interest in exploring microcombs outside this band. Such exploration helps specific applications and presents an avenue for innovative dispersion control techniques. Octave-spanning integrated microcombs with 1064 nm pumping have been shown in Si_3_N_4_ resonators^4,23^ and demonstrated for optical-clock division.^24^ However, these demonstrations have relied on traditional anomalous dispersion resonators with an increasing free spectral range (FSR) vs optical frequency. Soliton excitation in such resonators involves fast frequency sweeping with off-chip modulators^25^ to mitigate the thermal instabilities in the resonator occurring at the 100 ns scale that undermine comb formation.^26–28^ This excitation scheme poses limiting challenges, especially when considering the photonic integration of microcomb systems. In addition, such anomalous dispersion resonators typically facilitate bright soliton pulses, consisting of a pulse with enhanced peak power and a sech^2^ temporal profile riding atop the constant pump background. Finally, for short wavelength bands in the near IR and visible, anomalous dispersion becomes more challenging or impossible to generate by adjusting microresonator geometry.

Conversely, dark solitons, created in normal dispersion, consist of a distinct dark-pulse formation set against a backdrop of the constant pump at a similar intensity,^29–31^ offering higher pump-to-comb conversion efficiency and a pulse shape commensurate with a more flattop spectral envelope. However, these solitons occur in normal-dispersion resonators, with a decreasing FSR vs optical frequency. Phase matching in such resonators could be achieved with engineered mode crossings.^31,32^ However, as the ring waveguide width (RW) becomes the single parameter that simultaneously provides control of both dispersion and mode degeneracy in a resonator, there is less flexibility to design microcombs. An additional, partial degree of freedom could be created by introducing an auxiliary coupled resonator^7,33^ that alters the dispersion of the main comb-generating resonator by introducing a mode crossing. However, this approach provides limited dispersion control and induces side effects through wide-band resonator coupling. Moreover, the narrowing and more stringent coupling-gap requirements between coupled resonators pose fabrication challenges for pump wavelengths toward the visible.

A recent powerful approach that relies on photonic-crystal resonators (PhCR)^9,10,22^ allows the precise targeting of specific modes inside the resonator and the mode-by-mode construction of resonator meta-dispersion.^22^ The photonic-crystal modulation induces a photonic bandgap in a targeted mode, according to the perioicity of the modulation. It can, for example, support spontaneous soliton formation by shifting the pump mode toward a lower frequency.^9^

Here, we delve into the formation of dark-soliton microcombs at 1064 nm, facilitated by a photonic bandgap of the microresonator mode excited by the pump laser [Figs. 1(a) and 1(b)]. We fabricate the devices in our tantalum pentoxide (Ta_2_O_5_, hereafter tantala) on the SiO_2_ photonic platform, which provides nonlinear^34^ and linear^35^ photonics by exhibiting high linear and nonlinear coefficients and low losses across the visible to near-infrared spectrum. By varying the RW of the microresonator, we demonstrate control over the spectral bandwidth of the generated combs while preserving the bandgap that controls overall phase matching. Furthermore, we explore the comb existence range by means of numerical modeling and show that the comb exists for a wide range of experimentally controllable parameters [Fig. 1(d)]. Our results could be used in applications such as bioimaging and spectroscopy, where better light penetration is available at the 1–1.35 *μ*m spectral window,^36^ and for commodifying atomic quantum technologies by providing photonic integrated access to common optical atomic transitions^30^ such as Rb, CS, and Sr, located below 1 *μ*m.

We create PhCRs to induce the bandgap in a specific resonator mode. The inner wall of the PhCR contains an azimuthally uniform oscillation with periodicity πR/m, where R is the ring radius and m is the mode number of the pump mode. This RW oscillation induces a bandgap at the pump mode to fulfill the phase matching condition in normal-dispersion rings [see Fig. 1(a)]. Such split-mode pumping allows the manual tuning of the laser into the lower frequency resonance to generate the dark soliton [Fig. 1(b)]. Figure 1(b) shows a 200 GHz repetition rate soliton microcomb with a wide spectrum that spans 100 nm.

Figure 1(c) illustrates our experimental setup for soliton generation and characterization. We pump the PhCR with a continuous wave laser at 1064 nm using a ytterbium-doped fiber amplifier. Both backward and forward propagating soliton pulses can be generated in the PhCR, and the former usually has a higher power. Hence, we use a circulator before the PhCR chip to measure the backward propagating pulse with an optical spectrum analyzer. Part of the comb, extracted with the circulator, is coupled into a wavelength-division multiplexing (WDM) filter to separate the residual pump and other comb lines, which we monitor with an oscilloscope after photodetection.

We engineer the dispersion and tailor the comb spectrum by changing the PhCR geometry through the RW and altering the bandgap induced by the PhCR. We design for a soliton repetition rate of 200 GHz, which corresponds to a ring radius of 108 *μ*m. To quantify the effect of the dispersion, bandgap, and power on the comb spectrum, we turn to the normalized, modified Lugiato-Lefever Equations (LLE) that couple between the forward and backward propagation directions inside a Kerr resonator: $\partial_{\tau }\psi_{r/t}=-(1+i\alpha )\psi_{r/t}-\frac{i}{2}\beta \partial_{\theta }^{2}\psi_{r/t}+i\left({\left|\psi_{r/t}\right|}^{2}+2{\left({\left|\psi_{t/r}\right|}^{2}\right)}_{avg}\right)\psi_{r/t}+F-i\varepsilon_{PhC}\psi_{t/r,avg}$. Here, τ is time, θ is the resonator angular coordinate, $\psi_{t}$, and $\psi_{r}$ are the intraresonator fields in the forward and backward directions, respectively. $\frac{i}{2}\beta \partial_{\theta }^{2}\psi_{r/t}$ is the dispersion, ${\left|\psi_{\text{r}/\text{t}}\right|}^{2}\psi_{r/t}$ and $\left(2{\left({\left|\psi_{t/r}\right|}^{2}\right)}_{avg}\right)\psi_{r/t}$ are the Kerr nonlinearity contributions, F is the pump laser field with a frequency lower than the resonator mode by the detuning, which we label α, $\psi_{t/r,avg}$ is the azimuthally averaged field, and ${\text{ε}}_{PhC}$ is the bandgap induced pump mode shift.^22,37^ Notably, the normalized dispersion coefficient in the LLE is defined as $\beta =-2D_{2}/\kappa$, where $D_{2}/2\pi$ is the GVD coefficient and κ/2π is the half-linewidth of the resonator. We further define the integrated dispersion as $D_{int}(\mu )=\omega_{\mu }-\left(\omega_{p}+\mu \times D_{1}\right)=D_{2}\mu^{2}/2$, where $\omega_{p}$ is the pump-laser frequency, $D_{1}/2\pi$ is the resonator FSR near the pump-laser frequency, and μ is an integer indexing the resonances relative to the pumped mode μ=0. Figure 1(d) shows a sample comb existence range plot for different pump field values F at a normal GVD value of $D_{2}=-3\text{MHz}$.

To explore the existence range of the dark soliton in more detail, we solve the LLE for the backward and forward propagating fields for a wide range of detuning values. As the detuning increases, spectral horn features are formed,^10^ which are the equivalent of dispersive waves in anomalous GVD rings in that they represent spectral broadening. A further increase in pump detuning results in a frequency comb. With the simulations, we obtain the backward propagating comb power for each detuning value and assess performance based on the maximal value. We see that the smallest bandgap required to form a comb increases with increasing F. We attribute this to the more significant Kerr shift experienced by the pumped resonances with increased F, leading to a larger bandgap required to achieve phase matching. Increasing the F values further results in multiple solitons or noisy comb formation.

We perform single parameter sweeps to isolate the effects of $D_{2}$ and bandgap on the bandwidth of the formed combs (see Fig. 2). We set the field amplitude F = 3. As we sweep the bandgap [Fig. 2(a)], we see that for values beyond the threshold, a comb appears, and as we increase the bandgap, we obtain an increasing comb spectrum bandwidth. This shows that a larger bandgap facilitates phase matching with modes further away from the pump. As we increase the bandgap above 18 half-linewidths, phase matching does not occur.

Next, we set the bandgap to a constant value of ten half-linewidths and sweep the dispersion parameter $D_{2}$ [see Fig. 2(b)]. Notably, as the dispersion becomes less negative, showing a flatter dispersion curve, the same bandgap is sufficient to phase match more remote modes, form more distant horn features, and deliver wider combs.

To perform our experiments, we fabricate air-clad devices on the Ta_2_O_5_ platform, where a 570 nm tantala layer is used as the device layer. To characterize the PhCR modes in the 1064 nm band (Fig. 3), we couple light onto the chip via a lensed fiber and scan the laser across the lower frequency resonance of the split mode. We collect the output light with a lensed fiber and measure the spectrum with an OSA. The quality factors and dispersion of the resonances are measured by the use of a calibrated Mach–Zehnder interferometer technique^38,39^ for the fundamental transverse electric modes. We experimentally extract the integrated dispersion $D_{int\;}(\mu )$. Here, we do not include higher-order dispersion terms. The integrated dispersion shows a characteristic normal behavior, with a decreasing FSR with optical frequency [see Fig. 3(a)]. As the RW narrows, we can see that the dispersion becomes flatter, with smaller $D_{2}$ absolute values.

To measure resonator quality factors, we fit the measured resonances^40^ [Fig. 3(b)] and extract $Q_{i}$ and $Q_{c}$, the intrinsic and coupling quality factors, respectively. We achieve $Q_{i}$ in the range of 2 to 3 × 10^6^. We find that $Q_{i}$ increases with the RW. We attribute this to the intraresonator field being more contained within the waveguide, resulting in less scattering loss from the sidewall than narrower waveguides. Further increasing the RW beyond 2.25 *μ*m does not improve $Q_{i}$. The coupling quality factor $Q_{c}$ for the best-coupled devices with a nominal coupling gap of 275 nm is shown to be 8 × 10^6^ at a 281 THz pump frequency. Notably, $Q_{c}$ is RW independent, as we expected, and shows a linear slope as the frequency increases.

The induced bandgap results in the frequency splitting of the pump mode, and we control the magnitude of the mode splitting by altering the photonic crystal amplitude [APhC in Fig. 1(a)]. Figure 3(c) shows the measured mode splitting as a function of APhC in 200 GHz PhCRs. It is intuitively reasonable to see that the same APhC modulation depth imprinted on narrower waveguides (blue) results in more significant mode splitting. Sample mode splits are shown in Fig. 3(d).

Precise control of external coupling is essential for low threshold power and high pump-to-comb conversion efficiency. The threshold power for parametric oscillation is proportional to $4P_{th}∼1/Q_{i}^{2}\cdot {\left(1+K\right)}^{3}/K$, where $K=Q_{i}/Q_{c}$, resulting in a minimum for K = 0.5, which is the operation region we experimentally approach [see Fig. 1(b)]. Here, we use a pulley coupler in which we vary the gap and coupling length to obtain critical coupling. A more precise design is possible,^41^ which is especially useful for the outcoupling of broader comb spectra. We obtain nearly critical coupling in our chip for a coupling length of 30 *μ*m with a pulley coupler gap of 275 nm.

One notable drawback of pumping the PhC-modulation-induced split mode is elevated threshold power due to the energization of both forward and backward propagating modes, increasing the threshold power by up to a factor of 4 for critically coupled resonators.^42^ This can be alleviated by the use of pump recycling techniques^8^ or solved by employing recently introduced novel phase matching schemes.^43^ Alternatively, the backward reflection can be harnessed to enable self-injection locking of PhCR-coupled lasers and channel the power into the forward direction.^44^

Next, we explore soliton formation. We pump the PhCR with 20 dBm on-chip power and manually increase the laser detuning to selectively pump the split mode’s lower frequency component. We experimentally show that by controlling the dispersion together with the bandgap, we obtain broad control over the resulting comb bandwidth [see Fig. 4(a)].

To obtain a larger spectral bandwidth covering a wavelength range below 1000 nm and even further to potentially allow f-2f referencing, we must operate in a different dispersion regime. To achieve flatter dispersion, we pump rings with a narrower RW, which in turn results in a lower intrinsic quality factor and, hence, an increased $P_{th}∼1/Q_{i}^{2}$. In our experiments, the lensed fiber-to-chip coupling loss is ∼5 dB. Due to this sub-optimal facet coupling, we require higher power on the chip to form solitons. Future experiments that address this limitation could explore even broader bandwidth solitons, potentially covering the near-infrared relevant for Rb or Cs spectroscopy. Nevertheless, as the coupling into the chip and the sidewall waveguide roughness can be significantly improved, there is no fundamental factor limiting the bandwidth of the combs achievable with our approach.

Finally, it is known that solitons with certain parameters are prone to the formation of breathing instabilities,^45,46^ especially in normal-dispersion resonators.^29^ These are slow oscillations compared to the comb’s repetition rate, causing the fluctuation of the soliton spectrum in time and can be detrimental to some microcomb applications. The experimental signature of such breathers is an oscillatory peak on the electrical spectrum of the comb power, which we measure with an electric spectrum analyzer (ESA) [Fig. 4(b)]. We show here that by reducing the pump-cavity detuning after forming a soliton, we access an operating regime with zero breather amplitude, resulting in low relative intensity noise. We show [Fig. 4(b) that as the peak is detuned, the soliton spectrum retains the classic dark soliton spectrum with more defined enhancements at the edge of the spectrum (blue top) as opposed to the smoothed-out spectrum of the breather (magenta bottom).

In conclusion, we demonstrate 1064 nm dark-soliton microcombs in normal-dispersion PhCRs implemented in the Ta_2_O_5_ integrated photonics platform. The photonic-crystal-induced bandgap enables phase matching and soliton formation. The control of the RW and photonic crystal amplitude alters the dispersion and induced bandgap, respectively. These, in turn, determine the spectral bandwidth of the resulting frequency comb. We show that despite the appearance of the parasitic breather states, they could be removed by controlling the laser-cavity detuning. The efficiency and threshold power of our comb generation at 1064 nm can be further improved by designing better in-and out-couplers, utilizing pump recycling techniques^8^ demonstrated at the 1550 nm band, or employing novel phase matching schemes.^43^ Our work will impact applications at 1064 nm by providing chip-scale, low-power, and efficient frequency combs spanning biological imaging and crucial atomic transition windows.

## Figures and Tables

**FIG. 1.**
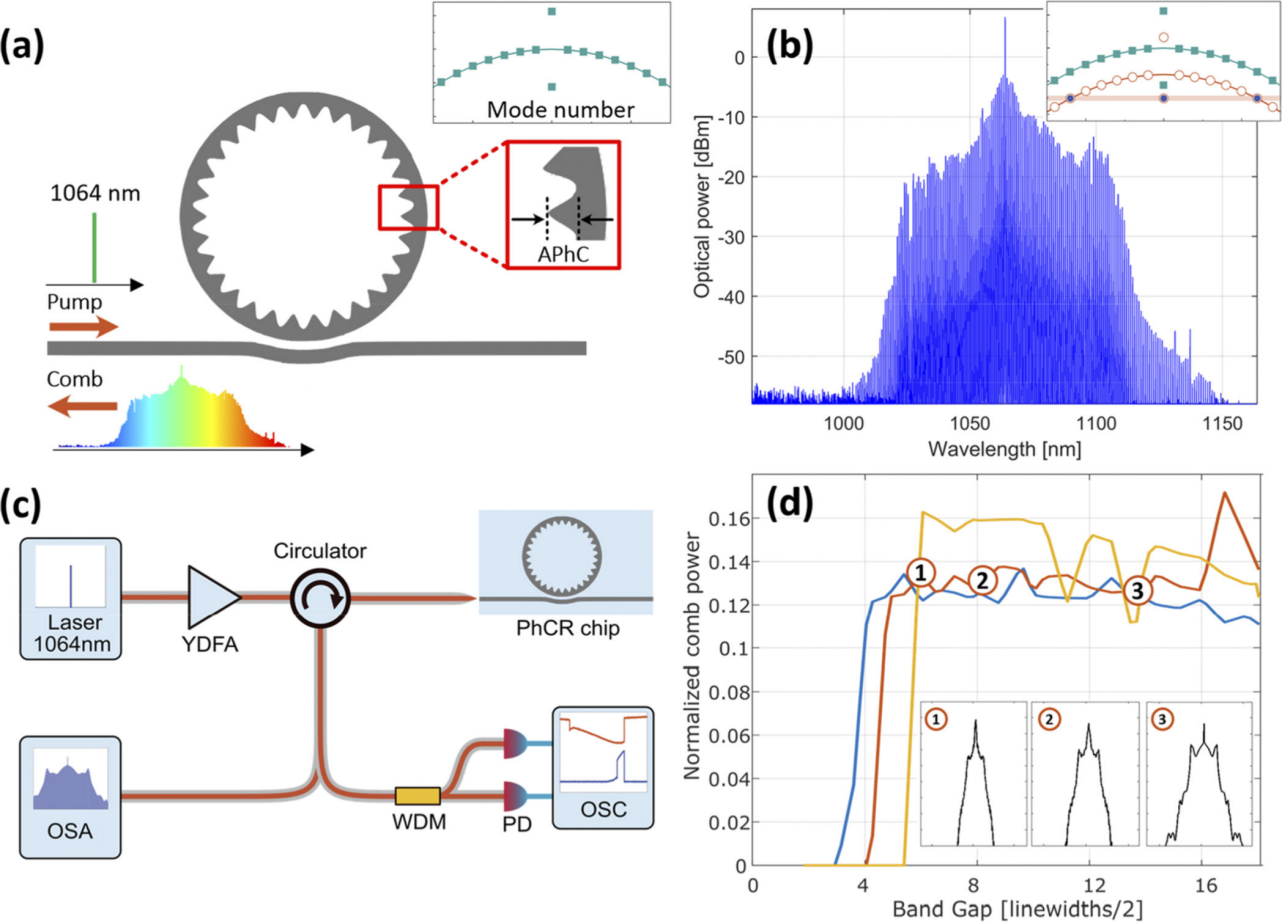
Generation of 1064 nm dark soliton microcombs. (a) Schematic of PhCR that generates a backward-propagating soliton. The top right inset shows the PhCR modes with normal dispersion and the pump mode split by the photonic crystal. (b) Soliton microcomb spectrum spanning 100 nm with a 200 GHz repetition rate. The spectrum is normalized to show on-chip power. The inset shows the phase-matching mechanism; the Kerr effect shifts the excited PhCR dispersion (red circles) while the pump mode is shifted by half the amount. (c) Experimental setup: The PhCR is pumped via a 1064 nm laser amplified with a ytterbium-doped fiber amplifier (YDFA) through a circulator. The backward propagating comb is collected and measured via an optical spectrum analyzer (OSA). A time-dependent measurement of the comb power and transmission is collected through a photodetector (PD) on an oscilloscope (OSC). (d) Modeled soliton existence range as a function of the photonic bandgap for different normalized pump field amplitudes F. The minimum required bandgap for soliton formation is shown to increase with F. The normalized comb power is calculated for a dispersion value of $D_{2}$ = −3 MHz and is taken for the detuning that results in maximal comb power. The insets show sample soliton spectra along the F = 3 line.

**FIG. 2.**
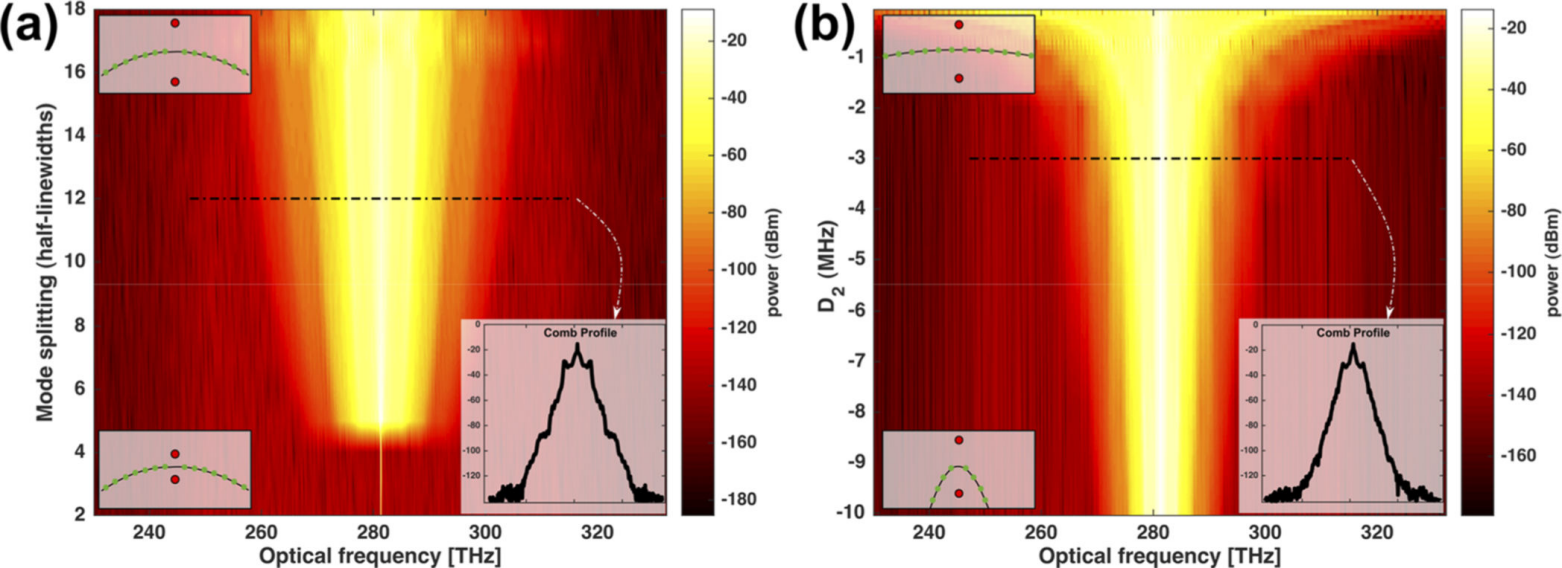
Modeling PhCR microcomb spectrum control. (a) Modeled comb power spectrum as a function of the bandgap that is controlled by the photonic crystal modulation amplitude. The model is calculated for F = 3 and normal dispersion $D_{2}=-3\text{MHz}$. The insets on the left illustrate the integrated dispersion curve $D_{int}$ remaining constant, with the split mode (red dots) being increased from bottom to top. (b) Modeled comb power spectrum as a function of $D_{2}$. The model is estimated for F = 3 and a photonic bandgap of ten half-linewidths. The insets on the left depict the integrated dispersion for large (bottom) and small (top) absolute values of $D_{2}$, with the bandgap remaining unchanged. The insets on the bottom right show the simulated comb profile for a bandgap of 12 linewidth (a) and dispersion $D_{2}=-3\text{MHz}$ (b).

**FIG. 3.**
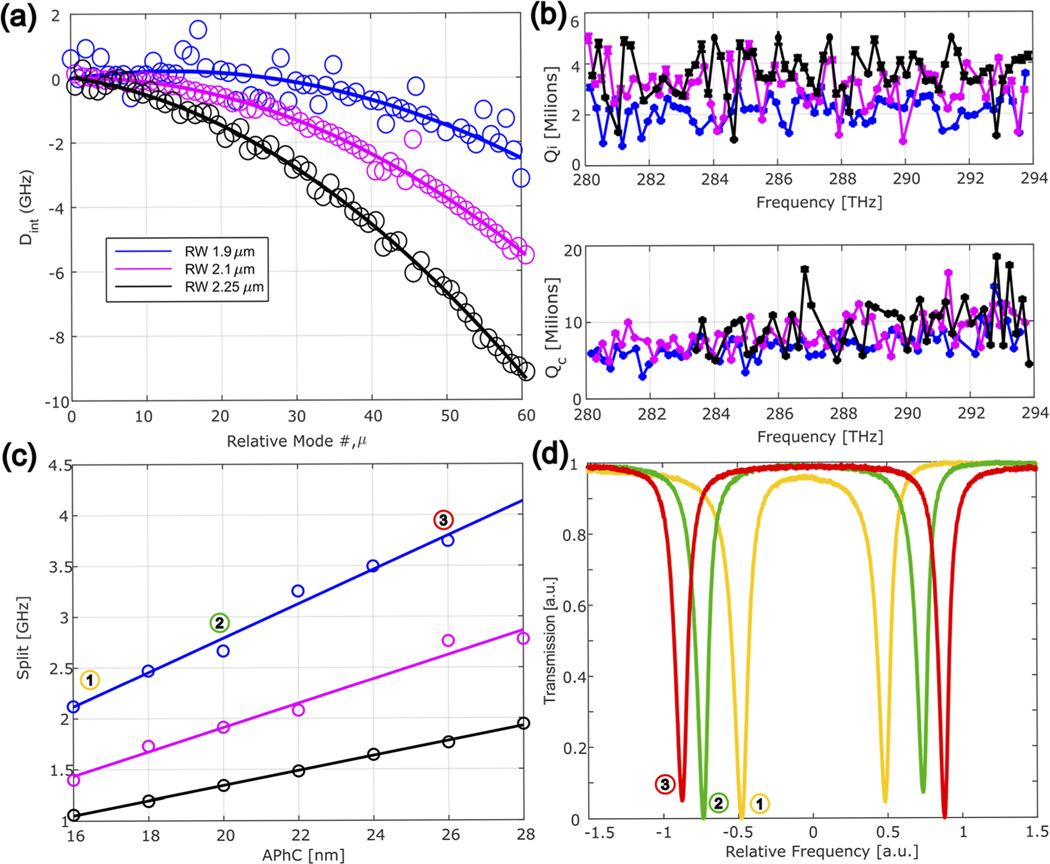
PhCR mode measurements in the 1064 nm band. (a) Measured $D_{int}$ for three different resonators RW, 1.9 *μ*m (blue), $D_{2}$ = (−2.5047 ± 0.3583) MHz, 2.1 *μ*m (magenta), $D_{2}$ = (−3.1072 ± 0.15) MHz, and 2.25 *μ*m (black), $D_{2}$ = (−3.9489 ± 0.2916) MHz, showing how the dispersion becomes more normal as the RW increases. (b) $Q_{i}$ (top) and $Q_{c}$ (bottom) as a function of optical frequency. The data in the top panel are taken with a large coupling gap (300 nm) to obtain a more accurate estimation of $Q_{i}$. (c) Measured mode split as a function of APhC. (d) Measured transmission of the split modes in the case with different APhC shown in (c). The color codes in (a)–(c) correspond to the same devices.

**FIG. 4.**
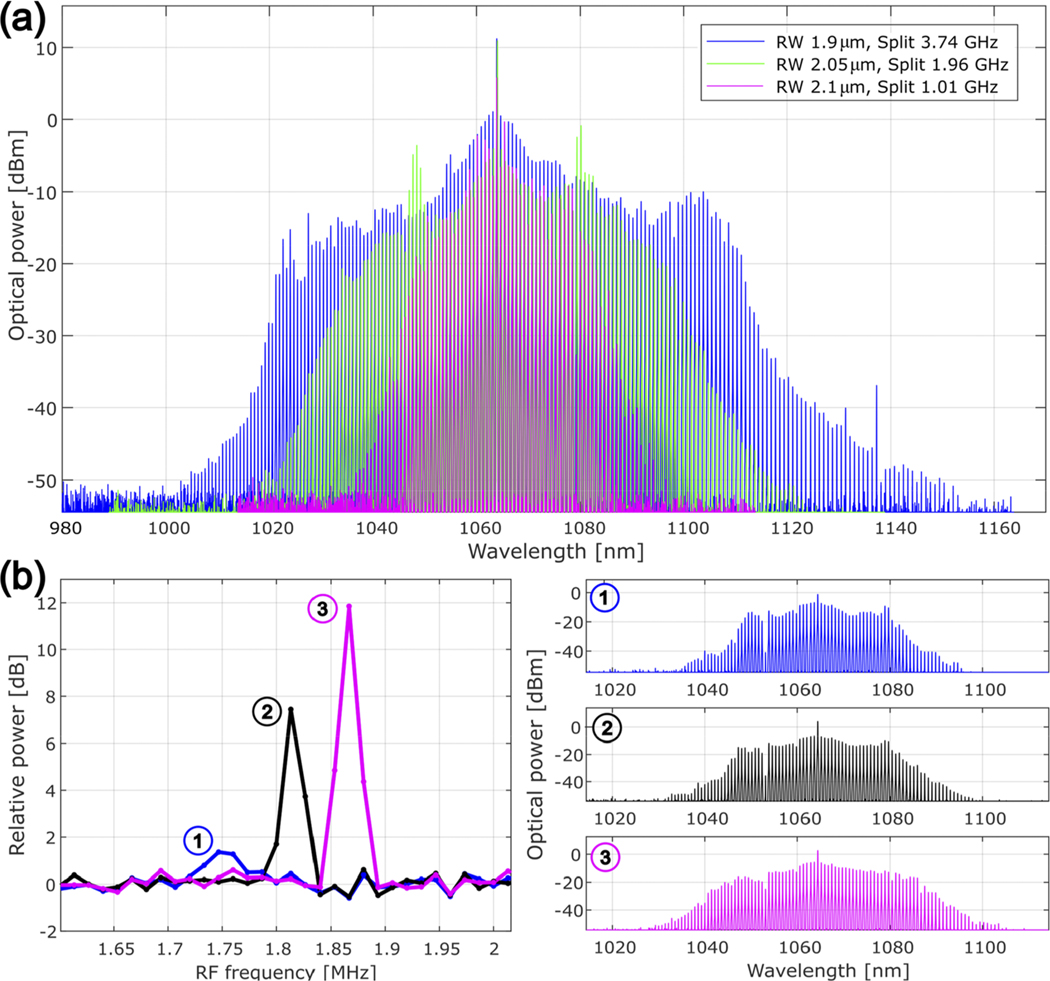
Bandwidth control of PhCR microcombs at 1064 nm. (a) Experimental spectra of combs with three different $D_{2}$ and photonic bandgap pairs, determining the bandwidth of the combs. The data are normalized to display on-chip comb power. (b) Tuning away the breather frequency shows how the comb spectrum changes as the breather is detuned.

## Data Availability

The data that support the findings of this study are available from the corresponding author upon reasonable request.
